# Self-Reported Red Eye Among Saudi Adults: Symptom Profile, Self-Management Practices, and Associated Factors

**DOI:** 10.3390/healthcare14193150

**Published:** 2026-09-22

**Authors:** Saja A. Almraysi, Dhiyaa A. H. Otayf, Mohammad A. Jareebi, Abdulaziz Ali Alagsam, Ghazi I. Al Jowf, Ahmed Y. Najmi, Rakan M. Hakami, Ali A. Dagreri, Eyad M. Albarrati, Mariam Zainab, Mutaz M. Zogail, Razan S. Hanafi, Omar Essa Mohammed Kirat, Ali Alabbas Ahmad Alhazmi, Farjah H. Algahtani

**Affiliations:** 1 Faculty of Medicine, Jazan University, Jazan 45142, Saudi Arabia; dhiyaaot@gmail.com (D.A.H.O.); ahmednajmiofficial@gmail.com (A.Y.N.); rak.alhakami@gmail.com (R.M.H.); aallii-23@hotmail.com (A.A.D.); eyad.albarrati@gmail.com (E.M.A.); moataz00999@gmail.com (M.M.Z.); 2 Department of Family and Community Medicine, College of Medicine, Jazan University, Jazan 45142, Saudi Arabia; 3 Ophthalmology Center, Prince Mohammed bin Nasser Hospital, Jazan Health Cluster, Jazan 82943, Saudi Arabia; aaalagsam@moh.gov.sa (A.A.A.); omarkirat911@gmail.com (O.E.M.K.); alhazmi1426@gmail.com (A.A.A.A.); 4 Department of Public Health, College of Applied Medical Sciences, University Medical Clinics Complex, King Faisal University, Al Hofuf 37912, Saudi Arabia; galjowf@kfu.edu.sa; 5 College of Medicine, Alfaisal University, Riyadh 11533, Saudi Arabia; mariamzainab1002@gmail.com; 6 King Fahad Armed Forces Hospital, Al Kurnaysh Br Rd, Al Andalus, Jeddah 23311, Saudi Arabia; razanhanafi@hotmail.com; 7 Oncology Center, Chair of Epidemiology and Public Health Research, Faculty of Medicine, King Saud University Medical City, King Saud University, Riyadh 12373, Saudi Arabia; falgahtani@ksu.edu.sa

**Keywords:** red eye, conjunctival hyperaemia, ocular surface disease, physical activity, Saudi Arabia, whitening eye drops

## Abstract

**Background/Objectives**: Red eye is a common ocular complaint, yet population data from Saudi Arabia are limited and largely confined to specific diagnostic entities. This study assessed the proportion of adults reporting red eye and described its clinical characteristics, perceived triggers, management practices, and associated factors. **Methods**: A cross-sectional survey was conducted between November 2025 and March 2026 among adults aged 18 years or older in Saudi Arabia, recruited by convenience sampling through social media. A structured self-administered questionnaire captured sociodemographic, systemic, ocular, and lifestyle characteristics and, among affected participants, symptom profile, triggers, and management. The outcome was ascertained with a single screening item, accompanied by a photograph to aid recognition, asking whether participants currently had, or had previously had, red eye. Multivariable logistic regression identified independently associated factors (*p* < 0.05). **Results**: Of 1670 responses, 1627 were analysed after exclusion of duplicates and implausible anthropometric entries. Current or previous red eye was reported by 474 participants (29.1%; 95% CI: 26.9–31.4). Most reported no or only slight effect on daily life (85.4%), and 76.8% of episodes resolved within 48 h. Independently associated factors were male sex (aOR = 1.76; 95% CI: 1.23–2.52; *p* = 0.002), cosmetic use around the eyes (aOR = 1.74; 95% CI: 1.20–2.53; *p* = 0.003), allergic rhinitis or sinusitis (aOR = 1.86; 95% CI: 1.38–2.52; *p* < 0.001), hypertension (aOR = 1.74; 95% CI: 1.04–2.91; *p* = 0.035), chronic anxiety disorder (aOR = 1.67; 95% CI: 1.09–2.55; *p* = 0.018), and higher education. Physical activity showed a graded inverse association (aOR = 0.57; 95% CI: 0.41–0.81 for the highest category). Only 27.6% consulted a physician, and 13.3% used whitening eye drops. **Conclusions**: Red eye was commonly recognised but usually infrequent, brief, and minimally disabling. As the outcome was a single unvalidated self-report item not bounded in time, these findings describe reported symptom burden rather than confirmed disease.

## 1. Introduction

Red eye is among the most frequent ocular complaints encountered in primary care and ophthalmic practice worldwide. It is a clinically heterogeneous presentation ranging from benign, self-limiting conditions to vision-threatening emergencies. The underlying aetiology may involve the conjunctival, episcleral, or scleral vasculature, with vasodilation and increased permeability arising from inflammatory, infectious, allergic, mechanical, or toxic mechanisms [1]. Although most cases are benign, pain, photophobia, visual disturbance, and corneal involvement warrant urgent assessment to exclude keratitis, uveitis, or acute angle-closure glaucoma [1,2].

Eye-related complaints account for approximately 2–5% of primary care consultations, with infectious conjunctivitis alone constituting nearly 35% of these presentations [1,2]. Globally, allergic conjunctivitis is estimated to affect 10–30% of the general population, with higher figures reported in symptom-based surveys than in clinically confirmed series [3,4,5,6]. Dry eye disease, another major contributor to ocular redness, has an age-adjusted prevalence of approximately 7.8% among women aged 50 years and older, and its burden is rising with increasing digital device use and population ageing [7]. In Saudi Arabia, a cross-sectional study reported allergic conjunctivitis in 18.3% of adults, with ocular redness present in 82% of affected individuals [8], while an investigation of microbial keratitis at a tertiary centre identified contact lens use, ocular surface disorders, and trauma as principal predisposing factors [9].

Despite this frequency, epidemiological data on red eye as a broad clinical presentation in Saudi Arabia remain limited, since most existing work has focused on specific diagnostic entities [8,9]. Behavioural and lifestyle contributors, the burden experienced by affected individuals, and the management practices they adopt have received little attention. Over-the-counter whitening (vasoconstrictor) eye drops are of particular interest: they are widely accessible without prescription and provide rapid cosmetic relief through adrenergic vasoconstriction, yet unsupervised use has been associated with tachyphylaxis, rebound hyperaemia, and masking of underlying pathology [10,11].

This study aimed to assess the proportion of adults in Saudi Arabia reporting red eye and to describe its clinical characteristics, perceived triggers, management practices, and associated factors. The study is a questionnaire-based survey of a symptom reported by participants rather than a clinically verified prevalence study, and the outcome is described throughout as self-reported red eye.

## 2. Materials and Methods

### 2.1. Study Design, Setting, and Sample Size

This cross-sectional study was conducted in Saudi Arabia between November 2025 and March 2026 among adults aged 18 years or older residing in the country. Participants were recruited by convenience sampling through an online self-administered questionnaire distributed via social media. No sampling frame, quotas, or stratification were applied. Individuals younger than 18 years and those who did not provide electronic informed consent were ineligible. Reporting follows the Strengthening the Reporting of Observational Studies in Epidemiology (STROBE) statement.

A target of 1500 participants was set a priori using the standard formula for a single proportion,

n_0_ = Z^2^ · *p* · (1 − *p*)/e^2^

where n_0_ is the required sample size, Z is the standard normal deviate for the desired confidence level (1.96 at 95%), *p* is the expected proportion (set at 0.50 to yield the maximum required sample size), and e is the desired precision. Because recruitment was by unrestricted social media convenience sampling rather than probability sampling, this figure describes the arithmetic precision of a proportion at that sample size and should not be read as a margin of error for the Saudi adult population; no design-based precision is claimed. The final analytical sample comprised 1627 participants.

### 2.2. Data Collection Tool

A structured self-administered questionnaire was developed in Arabic after review of the literature on red eye, ocular surface disorders, behavioural and environmental triggers, and management practices. Two rounds of pilot testing among the same 20 participants assessed clarity and comprehensibility; wording was refined between rounds, and pilot responses were excluded from analysis. An English version was also deployed. The instrument comprised five domains:

- Sociodemographic characteristics: Age, sex, nationality, residence, marital status, education, monthly household income, employment, weight, and height, with body mass index (BMI) calculated from self-reported values.
- Systemic diseases and lifestyle: Self-reported physician-diagnosed conditions, smoking status, and physical activity, the last assessed with a single item offering three options: none during the week, moderate or vigorous activity for less than 30 min on five days per week, and at least 30 min on five days per week.
- Ocular history: Previously diagnosed ocular conditions, vision correction, cosmetic use around the eyes, and ocular surgery, all self-reported.
- Red eye, among participants who reported it: Impact on daily life, recurrence, episode duration, associated symptoms, and perceived triggers.
- Management, among affected participants: Over-the-counter and prescription treatments, whitening eye drop use, and perceived improvement rated from 0 (no improvement) to 10 (very marked improvement).

The main outcome was ascertained with a single screening item accompanied by a photograph of a hyperaemic eye to aid recognition, asking whether participants currently had, or had previously had, red eye, with yes or no options. Red eye is a non-specific sign with many causes, and existing classifications address particular allergic and inflammatory conditions rather than the sign itself, so no established case definition was available to adopt [12,13,14]. The item applied no recall period and no frequency, duration, or severity threshold, so an affirmative response denotes current or previous ocular redness at any time. It has not been validated against clinical examination; although self-report agrees acceptably with administrative records for healthcare utilisation [15], that evidence does not extend to self-recognition of a clinical sign.

Participants answering yes were asked how often they had experienced red eye during the preceding six months (rarely, meaning less than once per month; several times per month; several times per week; daily or almost daily) and the typical duration of an episode (less than 48 h; less than one week; one week to four weeks; more than four weeks). These items characterised the reported episodes and did not contribute to case definition. Treatment options were not mutually exclusive, and the item did not distinguish between treatments used within a single episode, across episodes, or in sequence, nor record whether antibiotic or corticosteroid drops were prescribed, advised by a pharmacist, obtained without professional advice, or retained from an earlier prescription. The perceived improvement item was presented to all affected participants irrespective of their answers and therefore cannot be attributed to any individual treatment.

### 2.3. Data Collection, Processing and Quality Check

The questionnaire was distributed by a team of data collectors via WhatsApp, Telegram, Instagram, TikTok, X (formerly Twitter), and Snapchat, and any eligible adult encountering the link could participate voluntarily. It opened with an explanatory page describing objectives, confidentiality, voluntary participation, and participants’ rights, followed by a mandatory consent item preceding all study items. Every item was set as required, preventing submission of incomplete forms, and participants could respond only once. A total of 1670 responses were received; the number of individuals who viewed the link was not recorded.

Data were collected through three parallel Google Forms deployments, two in Arabic and one in English, exported separately and merged. The merged dataset was re-derived from the original exports and checked variable by variable against them, and three issues were addressed. First, 17 pairs of records shared an identical submission timestamp together with identical sex, nationality, age, weight, and height, indicating duplicate submissions arising during the merge; the second record of each pair was excluded. Second, records with biologically implausible anthropometry were excluded, defined as height below 130 cm or above 210 cm, weight below 30 kg or above 200 kg, or BMI below 12 or above 70 kg/m^2^. Such entries were treated as unreliable responses to a simple mandatory numeric item, casting doubt on the record as a whole, so whole records were excluded rather than BMI set to missing; because this is a judgement, a sensitivity analysis reinstated them with BMI omitted. Third, the response categories of the item on impact on daily life had been mislabelled during the merge, with the category corresponding to a slight effect assigned to the severe category; this was corrected against the original responses. No values were edited at any stage, and the remaining 64 analysed variables required no correction. The same survey also carried the Rosenberg Self-Esteem Scale, which was not an objective of the present study and is reported separately.

### 2.4. Statistical Analysis

Data were analysed in RStudio (R version 4.2.3; R Foundation for Statistical Computing, Vienna, Austria). Categorical variables were summarised as frequencies and percentages, continuous variables as means and standard deviations. The primary outcome was self-reported red eye, coded as a binary variable and analysed at participant level. A multivariable logistic regression model was fitted to identify independently associated factors. Candidate variables comprised sociodemographic characteristics, systemic diseases, lifestyle factors, ocular history, vision correction, cosmetic use, and ocular surgery, all specified a priori on grounds of clinical plausibility, with no data-driven selection. Ocular surface and eyelid margin conditions that themselves present with conjunctival hyperaemia were excluded from the primary model to avoid circularity.

Multicollinearity was assessed using variance inflation factors, and model adequacy by the ratio of outcome events to estimated parameters, exposed cell counts for each binary covariate, and Cook’s distance. Discrimination was summarised by the area under the receiver operating characteristic curve and Tjur’s R^2^, and calibration by the Hosmer–Lemeshow test and by inspection of observed against predicted risk across deciles of predicted probability. Crude odds ratios from univariable models are reported alongside adjusted estimates so that the magnitude and direction of confounding can be judged. Because 40 parameters were tested, *p*-values were additionally adjusted using the Benjamini–Hochberg false discovery rate procedure and reported as *q*-values. To address sparse-data bias, the model was refitted using Firth’s penalized maximum likelihood with profile penalized likelihood confidence intervals. Five sensitivity analyses refitted the model: excluding covariates with fewer than 20 exposed participants; adding the excluded ocular surface conditions as covariates; restricting the sample to participants without any such diagnosis; restricting the outcome to participants reporting recurrence at least several times per month during the preceding six months, so that outcome and exposures share a common recent time frame; and reinstating the records excluded for implausible anthropometry with BMI omitted. Significance was set at *p* < 0.05, and 95% confidence intervals are reported for all estimates.

### 2.5. Ethical Approval

This study was approved by the Standing Committee for Scientific Research at Jazan University, Saudi Arabia (Reference No. REC-47/05/1653, dated 3 November 2025). All procedures complied with the ethical standards of institutional and national research committees and with the principles of the 1964 Declaration of Helsinki and its subsequent amendments. Electronic informed consent was obtained from all participants before any data were collected.

### 2.6. Use of Artificial Intelligence

Generative AI was used solely for language editing. Study design, data collection, analysis, and interpretation were conducted entirely by the authors.

## 3. Results

### 3.1. Sociodemographic Characteristics

Of 1670 responses received, 17 were excluded as duplicate submissions and 26 for biologically implausible anthropometric entries, leaving 1627 for analysis (Figure 1). The mean age was 29 ± 13 years, and the mean BMI was 25 ± 5.8 kg/m^2^. Slightly over half were male (52.9%, n = 860), and most were Saudi nationals (82.7%, n = 1345) living in urban areas (87.5%, n = 1424). Nearly two-thirds were single (63.6%, n = 1035) and held a bachelor’s degree or diploma (66.9%, n = 1088). Students formed the largest occupational group (49.0%, n = 798), followed by employed participants (33.0%, n = 537). Monthly household income was distributed across the four bands, from 20.8% (n = 339) earning SAR 5000–9999 to 30.0% (n = 488) earning more than SAR 15,000 (Table 1).

### 3.2. Systemic Diseases and Lifestyle Characteristics

Allergic rhinitis or sinusitis was the most frequently reported systemic condition (19.2%, n = 313), followed by hypercholesterolaemia (10.9%, n = 178), chronic anxiety disorder (9.8%, n = 159), asthma (9.5%, n = 154), and depression (8.5%, n = 138). Hypertension and diabetes mellitus were reported by 6.9% (n = 112) and 6.5% (n = 105), respectively. Most participants had never smoked (79.9%, n = 1300). Physical activity was limited: 36.6% (n = 596) reported none during a typical week, 45.4% (n = 738) less than 30 min on five days per week, and 18.0% (n = 293) met the highest category (Table 2).

### 3.3. Ocular History and Reported Red Eye

Eyeglasses were used by 36.5% (n = 594) of participants and cosmetics around the eyes by 28.8% (n = 469). Soft contact lenses were used by 16.5% (n = 268), while rigid and scleral lenses were uncommon (2.6% and 2.8%). Chronic dry eye disease was the most frequently reported ocular surface condition (10.1%, n = 164), followed by periocular redness or inflammation (5.0%, n = 82) and allergic conjunctivitis (3.8%, n = 62). Refractive surgery had been undergone by 8.9% (n = 144); other ocular procedures were reported by fewer than 2% each (Table 3). Current or previous red eye was reported by 474 participants (29.1%; 95% CI: 26.9–31.4) (Figure 2).

### 3.4. Clinical Profile, Triggers, and Management of Red Eye

Among the 474 affected participants, functional impairment was limited: 48.9% (n = 232) reported no effect on daily life and 36.5% (n = 173) only a slight effect, while 11.0% (n = 52) reported a moderate and 3.6% (n = 17) a severe effect (Table 4). Over the preceding six months, recurrence was rarer than once per month in 63.9% (n = 303), several times per month in 18.4% (n = 87), several times per week in 11.6% (n = 55), and daily or almost daily in 6.1% (n = 29), and a typical episode resolved within 48 h for 76.8% (n = 364), while 1.5% (n = 7) reported episodes exceeding four weeks. The two dimensions were only loosely related: 53.2% reported both infrequent recurrence and episodes shorter than 48 h, whereas 3.0% reported recurrence at least several times per week together with episodes of one week or longer (Table 5). These distributions should be read with caution, since the screening item was not bounded in time while the recurrence item referred to the preceding six months, so participants whose most recent episode predated that window would fall into the lowest category.

Itching was the most common associated symptom (65.0%, n = 308), followed by burning or stinging (58.9%, n = 279), increased tearing (54.9%, n = 260), foreign body sensation (48.5%, n = 230), and blurred vision (46.6%, n = 221); discharge was least frequent (20.0%, n = 95) (Figure 3). Lack of sleep (80.4%, n = 381) and prolonged smartphone use (73.6%, n = 349) were the most frequently endorsed triggers, followed by eye rubbing (62.4%, n = 296) and allergy (57.8%, n = 274) (Figure 4).

Lubricating drops (63.9%, n = 303) and washing with water (58.9%, n = 279) were the most common management strategies, and watchful waiting was reported by 57.6% (n = 273). Antibiotic and corticosteroid drops were used by 20.0% (n = 95) and 11.0% (n = 52), respectively, and whitening eye drops by 13.3% (n = 63). Only 27.6% (n = 131) had consulted a physician. The treatment item was a grid requiring a yes or no answer for each of the seven options, so no response was missing: 224 participants (47.3%) combined watchful waiting with at least one active treatment, 49 reported watchful waiting alone, and 25 answered no to every option, indicating that they used none of the listed approaches. The mean perceived improvement score was 7.2 ± 2.7 on a 0–10 scale; because it was presented to all affected participants irrespective of their answers, it reflects overall symptom resolution rather than the effect of any single treatment.

### 3.5. Factors Associated with Red Eye

In the multivariable model, male sex (aOR = 1.76; 95% CI: 1.23–2.52; *p* = 0.002), a bachelor’s degree or diploma (aOR = 1.71; 95% CI: 1.28–2.28; *p* < 0.001), postgraduate education (aOR = 1.91; 95% CI: 1.11–3.29; *p* = 0.020), cosmetic use around the eyes (aOR = 1.74; 95% CI: 1.20–2.53; *p* = 0.003), allergic rhinitis or sinusitis (aOR = 1.86; 95% CI: 1.38–2.52; *p* < 0.001), hypertension (aOR = 1.74; 95% CI: 1.04–2.91; *p* = 0.035), and chronic anxiety disorder (aOR = 1.67; 95% CI: 1.09–2.55; *p* = 0.018) were independently associated with the outcome (Table 6). Physical activity showed a graded inverse association, with adjusted odds ratios of 0.74 (95% CI: 0.57–0.95; *p* = 0.017) for less than 30 min on five days per week and 0.57 (95% CI: 0.41–0.81; *p* = 0.002) for at least 30 min, compared with no weekly activity, and a significant test for linear trend (*p* < 0.001).

The model estimated 40 parameters for 474 outcome events, corresponding to 11.8 events per parameter. Variance inflation factors indicated no problematic multicollinearity, exposed cell counts fell below ten only for glaucoma surgery, strabismus surgery, and glaucoma, and no observation exceeded a Cook’s distance of 0.06. Discrimination was modest, with an area under the curve of 0.702 and a Tjur’s R^2^ of 0.105. The Hosmer–Lemeshow test gave *p* = 0.080, and observed against predicted risk agreed closely across the lower eight deciles, with divergence confined to the two highest-risk deciles, so calibration is adequate over the range in which most participants fall (Table S7).

After the Benjamini–Hochberg adjustment of all 40 *p*-values, five of the nine associations significant at the unadjusted threshold remained below a false discovery rate of 5%: allergic rhinitis or sinusitis (*q* = 0.002), a bachelor’s degree or diploma (*q* = 0.006), male sex (*q* = 0.019), the highest physical activity category (*q* = 0.019), and cosmetic use (*q* = 0.027). Postgraduate education, the lower physical activity category, chronic anxiety disorder (all *q* = 0.098), and hypertension (*q* = 0.157) did not meet that threshold. Crude and adjusted odds ratios are presented together in Table 6 and were similar for most covariates, but male sex showed marked confounding, with a crude odds ratio of 0.99 (95% CI: 0.80–1.23) against an adjusted 1.76, attributable chiefly to adjustment for cosmetic use, reported by 58.8% of women and 2.1% of men. Firth’s penalized estimation and five sensitivity analyses, covering sparse covariates, pre-existing ocular surface disease, a recent-onset outcome definition, and the anthropometric exclusions, all reproduced the principal associations; full results are provided in the Supplementary Materials (Tables S1–S6). Proportions are also reported by stratum so that readers may apply external population margins. The proportion reporting red eye was 31.3% at ages 18–24 (n = 890), 29.4% at 25–34 (n = 282), 27.1% at 35–44 (n = 203), 26.2% at 45–54 (n = 164), and 15.9% at 55 years or above (n = 88), and was almost identical in women (29.2%) and men (29.1%). It was 20.8% among participants with high school education or lower (n = 423), 32.2% among those with a bachelor’s degree or diploma (n = 1088), and 31.0% among those with postgraduate education (n = 116).

## 4. Discussion

### 4.1. Principal Findings

This cross-sectional study of 1627 adults profiles self-reported red eye in Saudi Arabia. Red eye was reported by 29.1% of participants, making it a commonly recognised symptom, although most episodes were infrequent, brief, and minimally disabling: 85.4% reported no or only slight interference with daily life, and the burden was concentrated in a small subgroup with recurrent or prolonged episodes. Multivariable analysis identified cosmetic use, allergic rhinitis or sinusitis, male sex, and a bachelor’s degree or diploma as independently associated factors that persisted after correction for multiple testing, while associations with hypertension, chronic anxiety disorder, postgraduate education, and physical activity were less robust.

### 4.2. Reported Frequency and Clinical Profile

The proportion reporting red eye (29.1%) is consistent with the literature describing red eye as a frequent ocular presentation, and exceeds condition-specific estimates from narrower case definitions. A Saudi study reported allergic conjunctivitis in 18.3% of adults, with ocular redness in 82% of those affected [8], while reviews estimate ocular allergy to affect 10–30% of the population, symptom-based assessments consistently exceeding confirmed diagnoses [3,4,5,6]. These estimates and the present outcome are not directly comparable. The outcome here is a single self-recognised symptom, unbounded in time and without frequency, duration, or severity criteria, capturing all causes of ocular redness rather than any single aetiology, and representing a lifetime or current report rather than a period prevalence.

The symptom profile reflects this breadth. Itching (65.0%), burning or stinging (58.9%), and increased tearing (54.9%) predominated, a mixed picture of allergic and ocular surface irritation. Blurred vision was reported by 46.6%; while this may reflect transient tear film disturbance, it warrants assessment when persistent or accompanied by pain [1]. Most episodes resolved within 48 h, but 6.1% reported daily or almost daily recurrence and 6.5% episodes exceeding one week. This subgroup, rather than the sample as a whole, carries the clinically meaningful burden.

### 4.3. Perceived Triggers and Care-Seeking

Lack of sleep (80.4%) and prolonged digital device use (73.6%) were the most commonly endorsed triggers, followed by eye rubbing (62.4%) and allergy (57.8%). These are attributions made by affected participants only, and should be read as perceived triggers rather than measured exposures. Their prominence is consistent with evidence linking sleep deprivation and screen use to ocular surface dysfunction through reduced blink rate, tear film instability, and increased inflammatory mediator expression [16,17]. Eye rubbing is a recognised mechanical stressor and a perpetuating factor in allergic conjunctivitis and keratoconus [18].

Most affected participants (72.4%) did not seek medical consultation. Read alongside the symptom profile, this is less a gap in care than a proportionate response, since the great majority reported mild, self-limiting episodes, and it is consistent with the literature on self-medication in ocular conditions [19]. The concern lies not in the consultation rate but in specific practices: antibiotic and corticosteroid drops were used by 20.0% and 11.0% respectively, and 13.3% used whitening (vasoconstrictor) drops, a practice associated with tachyphylaxis, rebound hyperaemia, and masking of underlying pathology [10,11].

### 4.4. Cosmetic Use and the Ocular Surface

Cosmetic use around the eyes was independently associated with red eye (aOR = 1.74; 95% CI: 1.20–2.53; *p* = 0.003), and the association strengthened when pre-existing ocular surface disease was adjusted for (aOR = 2.02) and when participants carrying such diagnoses were excluded (aOR = 2.04), arguing against an artefact of underlying disease. Eyeliners and mascara may contribute to Meibomian gland orifice obstruction, tear film instability, and direct chemical or allergic irritation [20,21,22].

### 4.5. Systemic and Psychological Factors

Allergic rhinitis or sinusitis showed the strongest systemic association (aOR = 1.86; 95% CI: 1.38–2.52; *p* < 0.001), which was mechanistically supported by the anatomical and immunological continuity between the nasal mucosa and the ocular surface via the nasolacrimal duct and by shared atopic pathways. Allergic rhinitis elevates histamine and inflammatory mediator levels, producing vasodilation and conjunctival hyperaemia, while concurrent itching promotes eye rubbing, itself a commonly perceived trigger in this sample [23,24]. Given that 19.2% of participants reported allergic rhinitis or sinusitis, management of concurrent allergic disease is an important component of red eye care.

Hypertension was associated with higher odds of red eye (aOR = 1.74; 95% CI: 1.04–2.91), and more strongly when the outcome was restricted to recurrent episodes (aOR = 2.89). A systematic review of dry eye syndrome reported comparable associations with hypertension, diabetes, and cardiovascular disease, indicating that systemic vascular and inflammatory conditions are relevant to ocular surface symptoms [25]. Chronic anxiety disorder was also associated (aOR = 1.67; 95% CI: 1.09–2.55). Anxiety is linked to sleep disturbance and elevated corticosteroid and catecholamine levels, both of which affect tear film stability [17,26], while autonomic dysregulation may contribute to conjunctival vascular reactivity [27]. Neither association survived correction for multiple testing, and these pathways were not measured here, so both remain interpretive.

### 4.6. Physical Activity

Physical activity showed a graded inverse association, with adjusted odds ratios of 0.74 for the lower and 0.57 for the higher activity category. This finding is presented as exploratory rather than as a principal result. The measure was a single crude self-report item, the outcome was unbounded in time, the design was cross-sectional, the association attenuated towards the null when the outcome was restricted to participants with recurrent episodes, and only the highest activity category survived correction for multiple testing. No mechanistic interpretation is warranted. The most plausible reading is that physical activity acts as a proxy for general health status and for correlated circumstances and behaviours that were not fully captured. Although reviews of exercise and ocular health describe reductions in oxidative stress and inflammatory mediators within ocular tissues [28], the present data can neither support nor test such pathways.

### 4.7. Demographic and Educational Factors

Male sex was independently associated with red eye (aOR = 1.76; 95% CI: 1.23–2.52; *p* = 0.002). Sex-related differences in ocular surface symptoms have been reported previously and may reflect care-seeking behaviour, occupational and environmental exposures, and hormonal influences on ocular surface biology [29]. Higher education was also associated with the outcome, for both bachelor-level (aOR = 1.71) and postgraduate (aOR = 1.91) participants. Education is unlikely to affect ocular surface health directly; the association is more plausibly a marker of correlated exposures and reporting behaviour, since more highly educated participants may spend longer on digital screens and be readier to recognise and report a previous episode.

### 4.8. Implications, Strengths, and Limitations

The associations differ in the strength of evidence supporting them. Allergic rhinitis or sinusitis, cosmetic use, male sex, a bachelor’s degree or diploma, and the highest physical activity category remained below a 5% false discovery rate after adjustment for 40 tests, and each was stable under Firth’s penalisation. Hypertension, chronic anxiety disorder, postgraduate education, and the lower physical activity category did not survive correction and should be regarded as hypothesis-generating.

Clinically, the association with allergic rhinitis supports evaluation extending beyond symptomatic treatment to management of underlying allergic disease, and the association with cosmetic use supports structured patient education on safe cosmetic practice. From a public health perspective, the combination of low consultation rates with reported use of antibiotic, corticosteroid, and whitening drops is the most actionable finding. The survey did not record whether these treatments were prescribed, recommended by a pharmacist, or obtained without professional advice, so neither their appropriateness nor their source can be determined. What can be said is that a substantial minority reported using agents that require clinical judgement, which argues for targeted awareness campaigns on appropriate eye drop use and on the features that warrant assessment.

The study has several strengths. The sample of 1627 adults yielded precise estimates, and the dataset was complete for every analysed variable. Candidate variables were specified a priori, and manifestations of ocular redness were excluded from the primary model to avoid circularity; sensitivity analyses adding those conditions and excluding participants carrying them confirmed the stability of the principal associations. The analysis dataset was re-derived from the original survey exports and verified variable by variable, and participant-level data are available so that every reported figure can be reproduced.

Several limitations apply. Most importantly, the outcome relied on a single self-report screening item not validated against clinical examination; its sensitivity, specificity, test–retest reliability, and agreement with ophthalmic assessment are unknown. The accompanying photograph and follow-up items were intended to reduce misclassification but do not constitute validation. The item applies no frequency, duration, severity, or recall criterion, so it groups a single remote episode with current recurrent symptoms, and because the screening and recurrence items cover different time frames, the recurrence distribution should be read with caution. Reported ocular and systemic diagnoses rest on participant recall. The cross-sectional design and unbounded outcome preclude temporal ordering, so no causal inference is warranted. Recruitment was by social media convenience sampling, and because sampling was non-probabilistic the confidence intervals describe sampling precision only; no post-stratification weights were applied, since weighting assumes selection is ignorable within cells and would not address selection on unmeasured characteristics such as interest in eye health. Stratum-specific proportions are therefore reported so that readers may apply external margins. Making all items mandatory eliminated missing data but may have prompted approximate answers where participants were uncertain. Perceived triggers were drawn from a fixed list of seven factors without a free-text option, the model explains only a small proportion of the variation, and residual confounding from unmeasured exposures including sleep and screen time cannot be excluded.

## 5. Conclusions

More than one quarter of the adults surveyed reported current or previous red eye, most often as infrequent, short-lived episodes with little or no effect on daily life. Cosmetic use around the eyes, allergic rhinitis or sinusitis, male sex, and a bachelor’s degree or diploma were independently associated with the outcome and remained so after correction for multiple testing, while associations with hypertension, chronic anxiety disorder, postgraduate education, and physical activity were less robust and are reported as hypothesis-generating. Low rates of professional consultation alongside reported use of antibiotic, corticosteroid, and whitening eye drops highlight a need for public education on appropriate eye drop use and on recognising symptoms that warrant assessment. Because the outcome is a single unvalidated self-report item that is not bounded in time, these findings describe reported symptom burden rather than clinically confirmed disease, and the associations are cross-sectional and not causal. Prospective studies using a validated instrument, clinical examination, and standardised objective hyperaemia grading are recommended.

## Figures and Tables

**Figure 1:**
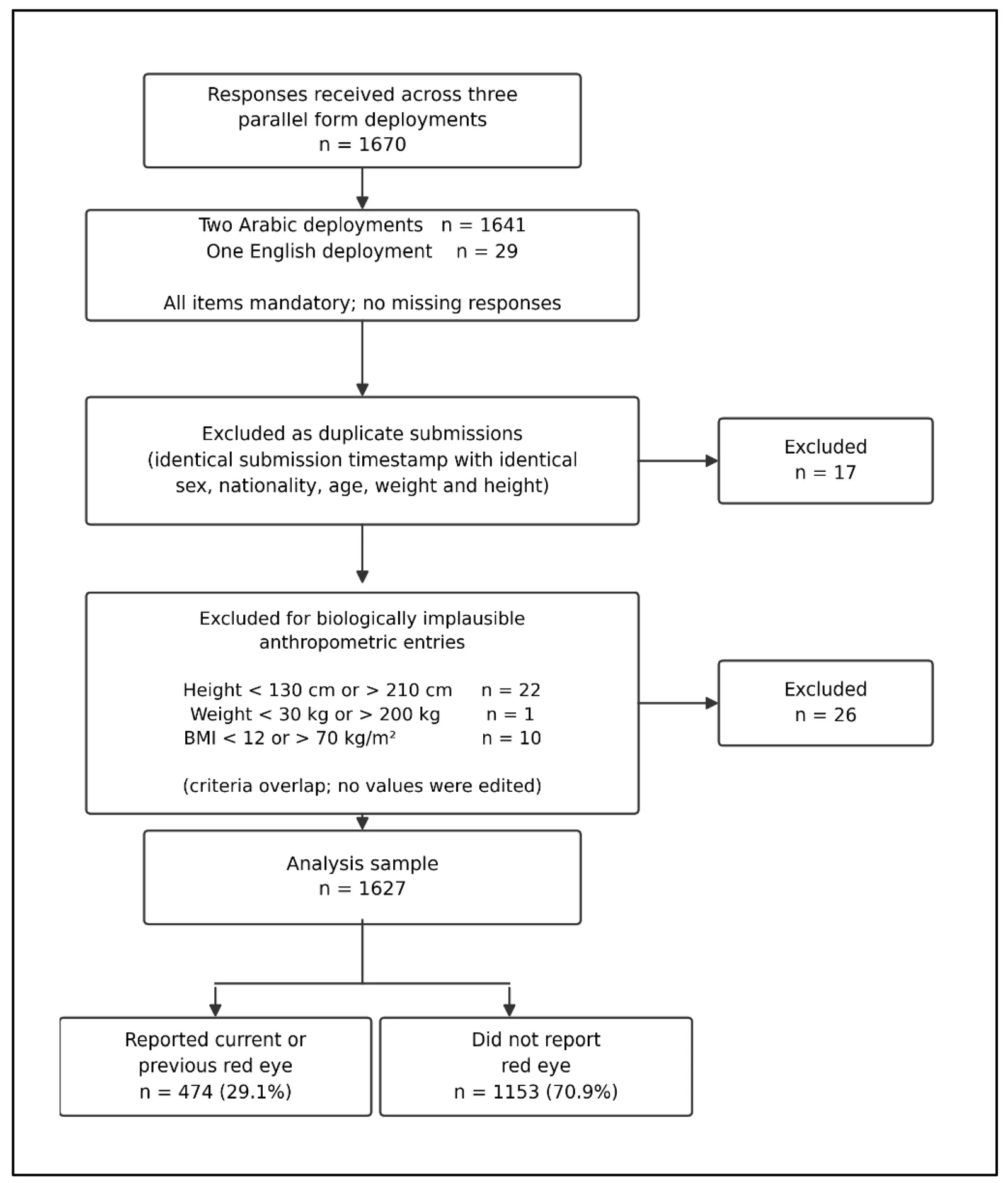
Flow of survey responses from collection to analysis.

**Figure 2:**
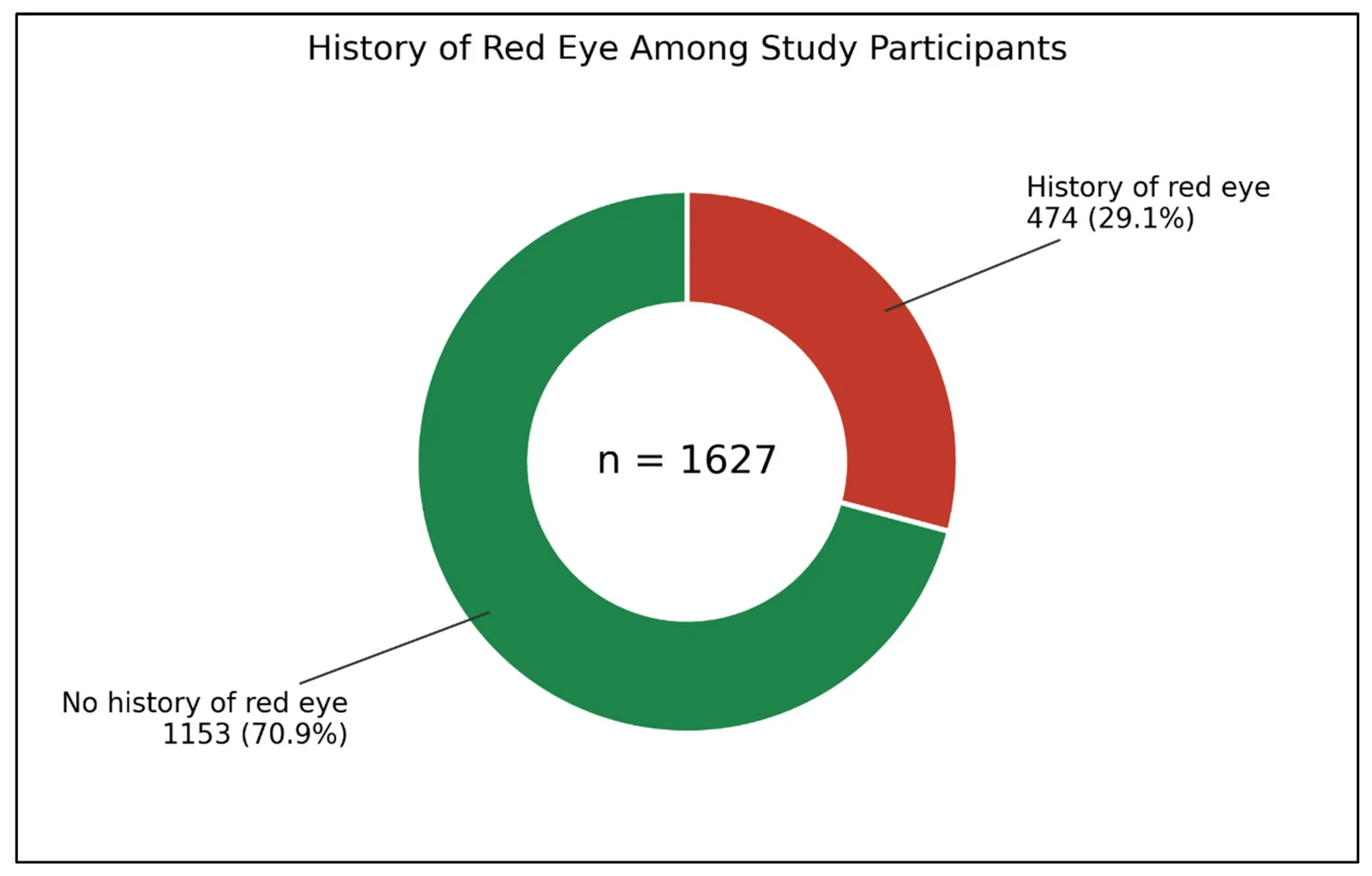
Proportion of participants reporting current or previous red eye (n = 1627). The red segment represents participants reporting current or previous red eye (n = 474; 29.1%), and the green segment those reporting no history of red eye (n = 1153; 70.9%).

**Figure 3:**
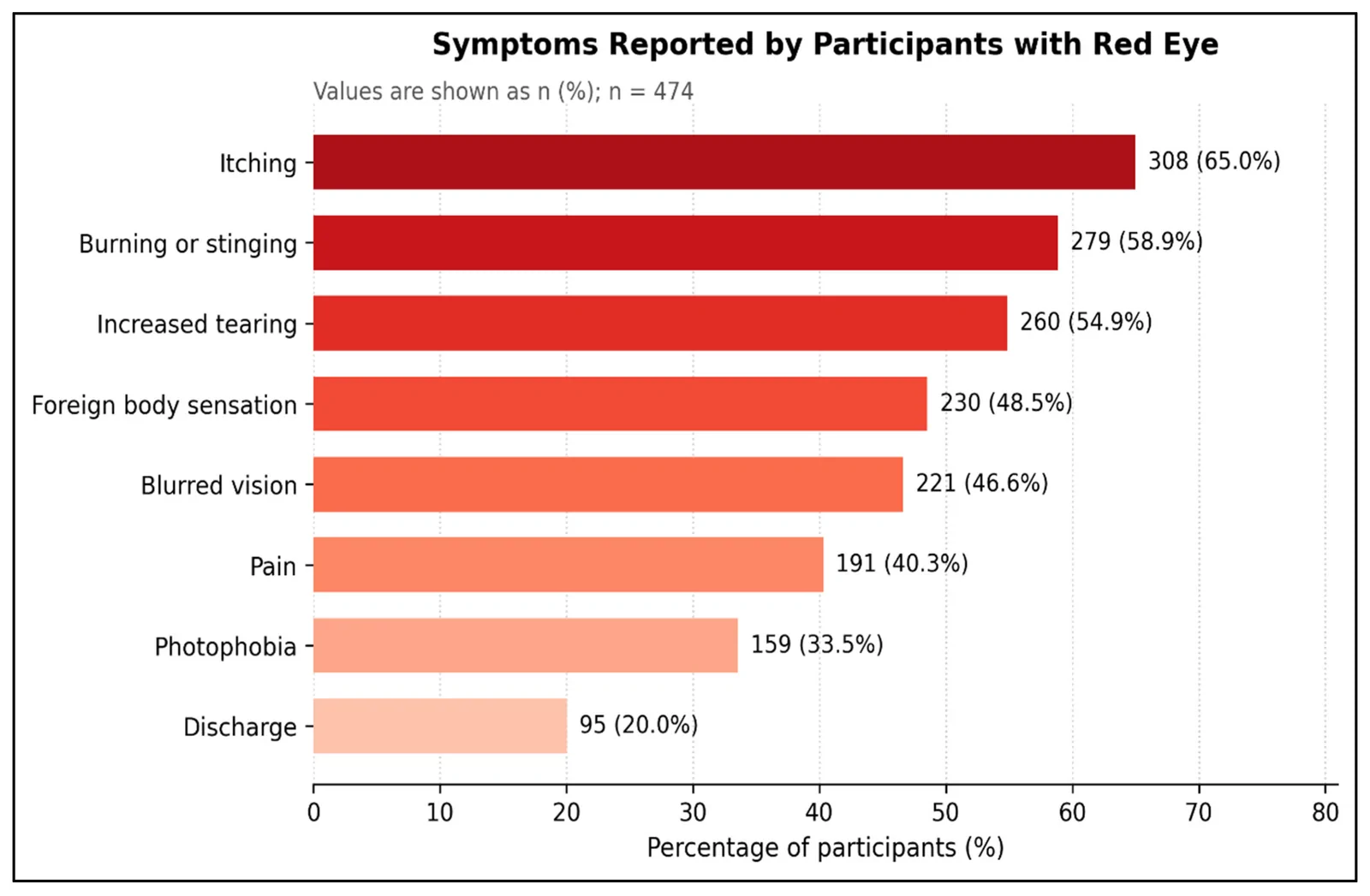
Frequency of symptoms reported by participants with red eye (n = 474).

**Figure 4:**
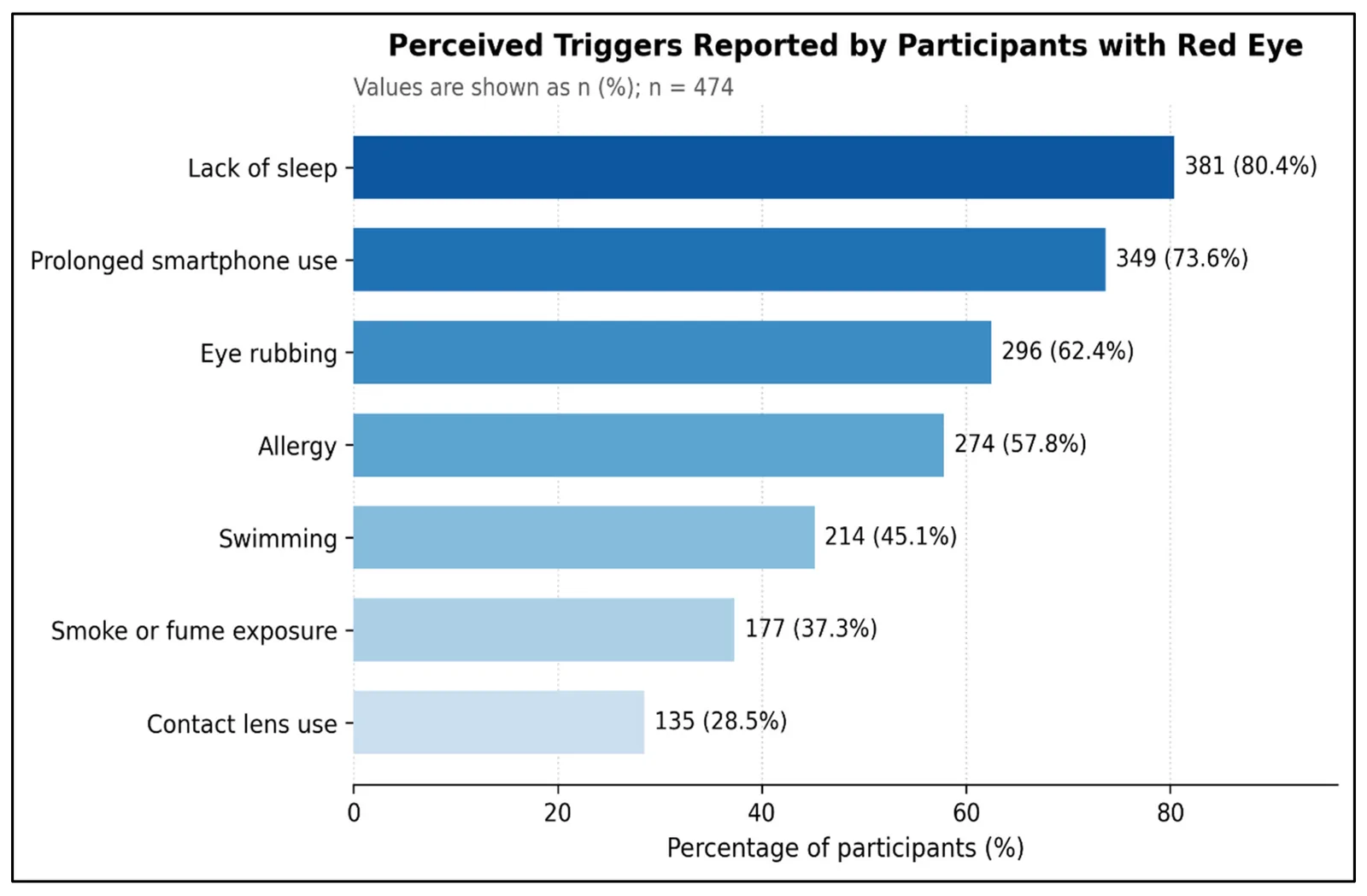
Frequency of perceived triggers reported by participants with red eye (n = 474).

**Table 1:** Sociodemographic characteristics of the study participants (n = 1627).

Characteristics	Mean ± SD/Frequency (%)
Age (years)	29 ± 13
BMI (kg/m^2^)	25 ± 5.8
**Sex**	
Female	767 (47.1%)
Male	860 (52.9%)
**Nationality**	
Saudi	1345 (82.7%)
Non-Saudi	282 (17.3%)
**Residence**	
Urban	1424 (87.5%)
Rural	203 (12.5%)
**Marital status**	
Single	1035 (63.6%)
Married	531 (32.6%)
Divorced or widowed	61 (3.7%)
**Education level**	
High school or lower	423 (26.0%)
Bachelor’s degree or diploma	1088 (66.9%)
Postgraduate studies	116 (7.1%)
**Monthly household income (SAR)**	
Less than SAR 5000 (<~$1333)	444 (27.3%)
SAR 5000–9999 (~$1333–$2666)	339 (20.8%)
SAR 10,000–14,999 (~$2667–$3999)	356 (21.9%)
More than SAR 15,000 (>~$4000)	488 (30.0%)
**Employment status**	
Student	798 (49.0%)
Employed	537 (33.0%)
Unemployed	292 (17.9%)

***Abbreviations:*** *n: sample size; SD: standard deviation; BMI: body mass index; SAR: Saudi Arabian Riyal. USD equivalents are approximate, based on an exchange rate of 1 SAR = 0.267 USD.*

**Table 2:** Systemic diseases and lifestyle characteristics of the study participants (n = 1627).

Characteristics	Frequency (%)
**Systemic diseases**	
Allergic rhinitis or sinusitis	313 (19.2%)
Hypercholesterolaemia	178 (10.9%)
Chronic anxiety disorder	159 (9.8%)
Asthma	154 (9.5%)
Depression	138 (8.5%)
Hypertension	112 (6.9%)
Diabetes mellitus	105 (6.5%)
Hypothyroidism	77 (4.7%)
Rheumatoid arthritis	67 (4.1%)
Hyperthyroidism	41 (2.5%)
Autoimmune diseases	36 (2.2%)
**Physical activity**	
No weekly physical activity	596 (36.6%)
<30 min moderate/vigorous activity, 5 days/week	738 (45.4%)
≥30 min moderate/vigorous activity, 5 days/week	293 (18.0%)
**Smoking status**	
Never smoker	1300 (79.9%)
Current smoker	191 (11.7%)
Ex-smoker	136 (8.4%)

***Abbreviations:*** *n: sample size.*

**Table 3:** Ocular history among the study participants (n = 1627).

Characteristics	Frequency (%)
**Ocular conditions**	
Chronic dry eye disease	164 (10.1%)
Periocular redness or inflammation	82 (5.0%)
Allergic conjunctivitis	62 (3.8%)
Atopic dermatitis of the eyelids	45 (2.8%)
Chronic blepharitis	44 (2.7%)
Keratitis	42 (2.6%)
Glaucoma	26 (1.6%)
Facial nerve palsy	27 (1.7%)
**Vision correction**	
Eyeglasses	594 (36.5%)
Soft contact lenses	268 (16.5%)
Scleral contact lenses	45 (2.8%)
Rigid contact lenses	43 (2.6%)
**Cosmetic practices**	
Cosmetic use around the eyes	469 (28.8%)
**Ocular surgical history**	
Refractive surgery	144 (8.9%)
Strabismus surgery	23 (1.4%)
Cataract surgery	21 (1.3%)
Glaucoma surgery	17 (1.0%)

***Abbreviations:*** *n: sample size.*

**Table 4:** Clinical characteristics and management of red eye among affected participants (n = 474).

Characteristics	Frequency (%)/Mean ± SD
**Impact of red eye on daily activities**	
Did not affect	232 (48.9%)
Affected severely	17 (3.6%)
Affected moderately	52 (11.0%)
Affected slightly	173 (36.5%)
**Recurrence of red eye (preceding six months)**	
Rarely (less than once per month)	303 (63.9%)
Several times per month	87 (18.4%)
Several times per week	55 (11.6%)
Daily or almost daily	29 (6.1%)
**Typical duration of an episode**	
Less than 48 h	364 (76.8%)
Less than one week	79 (16.7%)
One week to four weeks	24 (5.1%)
More than four weeks	7 (1.5%)
**Medical consultation for red eye**	
Yes	131 (27.6%)
No	343 (72.4%)
Management practices *	
Lubricating eye drops	303 (63.9%)
Eye washing with water	279 (58.9%)
Watchful waiting (no active treatment)	273 (57.6%)
Warm or cold compresses	205 (43.2%)
Allergy medications	120 (25.3%)
Antibiotic eye drops	95 (20.0%)
Corticosteroid eye drops	52 (11.0%)
**Whitening eye drop use**	
Yes	63 (13.3%)
No	411 (86.7%)
Perceived treatment improvement score **	
Mean score	7.2 ± 2.7

*Abbreviations: n: sample size; SD: standard deviation*; * Percentages sum to more than 100% as participants could select multiple options. ** Perceived improvement score rated on a numerical scale from 0 (no improvement) to 10 (very marked improvement); reported for all participants who completed this item.

**Table 5:** Joint distribution of reported recurrence during the preceding six months and typical episode duration among participants reporting red eye (n = 474).

Recurrence (Preceding Six Months)	Less than 48 h	Less than One Week	One Week to Four Weeks	More than Four Weeks	Total
Rarely (less than once per month)	252 (53.2%)	39 (8.2%)	9 (1.9%)	3 (0.6%)	303 (63.9%)
Several times per month	63 (13.3%)	19 (4.0%)	5 (1.1%)	0 (0.0%)	87 (18.4%)
Several times per week	31 (6.5%)	17 (3.6%)	7 (1.5%)	0 (0.0%)	55 (11.6%)
Daily or almost daily	18 (3.8%)	4 (0.8%)	3 (0.6%)	4 (0.8%)	29 (6.1%)
Total	364 (76.8%)	79 (16.7%)	24 (5.1%)	7 (1.5%)	474 (100.0%)

*Percentages are of all 474 participants reporting red eye. Both items were asked only of participants who answered yes to the screening item and did not contribute to case definition. The screening item was not bounded in time, whereas the recurrence item referred to the preceding six months.*

**Table 6:** Multivariable logistic regression analysis of factors associated with self-reported red eye (n = 1627).

Associated Factor	Crude OR (95% CI)	aOR	95% CI	*p*-Value	*q*-Value (BH-FDR)
**Age**	0.99 (0.98–0.99)	0.99	0.97–1.00	0.120	0.338
**Sex (reference: Female)**					
**Male**	**0.99 (0.80–1.23)**	**1.76**	**1.23–2.52**	**0.002**	**0.019**
**BMI (kg/m^2^)**	1.01 (0.99–1.03)	1.02	1.00–1.04	0.127	0.338
**Nationality (reference: Non-Saudi)**					
**Saudi**	0.79 (0.60–1.04)	0.75	0.54–1.03	0.074	0.277
**Marital status (reference: Single)**					
**Married**	0.75 (0.59–0.95)	1.03	0.68–1.56	0.877	0.933
**Divorced or widowed**	0.59 (0.32–1.11)	0.95	0.43–2.10	0.902	0.933
**Residence (reference: Rural)**					
**Urban**	0.95 (0.69–1.31)	0.91	0.64–1.29	0.591	0.807
**Education level (reference: High school or lower)**					
**Bachelor’s degree or diploma**	**1.81 (1.38–2.36)**	**1.71**	**1.28–2.28**	**<0.001**	**0.006**
**Postgraduate studies**	1.71 (1.08–2.71)	1.91	1.11–3.29	0.020	0.098
**Employment status (reference: Unemployed)**					
**Employed**	1.07 (0.77–1.48)	0.98	0.66–1.45	0.933	0.933
**Student**	1.50 (1.11–2.04)	1.42	0.94–2.14	0.099	0.329
**Monthly household income (reference: SAR < 5000)**					
**SAR 5000–9999**	1.03 (0.75–1.40)	1.05	0.75–1.48	0.774	0.887
**SAR 10,000–14,999**	0.80 (0.58–1.11)	0.83	0.58–1.17	0.287	0.546
**SAR > 15,000**	1.28 (0.97–1.69)	1.16	0.85–1.59	0.345	0.574
**Smoking status (reference: Never smoker)**					
**Current smoker**	1.22 (0.88–1.69)	1.12	0.77–1.63	0.559	0.799
**Ex-smoker**	0.96 (0.65–1.43)	1.10	0.71–1.69	0.679	0.849
**Vision correction**					
**Eyeglasses**	1.61 (1.30–2.01)	1.25	0.98–1.61	0.076	0.277
**Soft contact lenses**	1.87 (1.42–2.45)	1.32	0.91–1.91	0.148	0.370
**Rigid contact lenses**	3.51 (1.90–6.50)	1.14	0.47–2.76	0.773	0.887
**Scleral contact lenses**	3.81 (2.08–6.98)	1.50	0.65–3.45	0.344	0.574
**Cosmetic use around the eyes (reference: No)**					
**Yes**	**1.71 (1.36–2.15)**	**1.74**	**1.20–2.53**	**0.003**	**0.027**
**Ocular surgical history**					
**Refractive surgery**	1.24 (0.86–1.79)	0.94	0.62–1.44	0.777	0.887
**Cataract surgery**	2.72 (1.15–6.44)	0.16	0.02–1.49	0.108	0.333
**Glaucoma surgery**	11.67 (3.34–40.79)	3.67	0.49–27.60	0.207	0.436
**Strabismus surgery**	5.72 (2.34–13.99)	1.78	0.54–5.90	0.345	0.574
**Ocular conditions**					
**Glaucoma**	4.73 (2.09–10.68)	1.07	0.27–4.26	0.921	0.933
**Facial nerve palsy**	4.25 (1.93–9.36)	1.75	0.48–6.40	0.395	0.615
**Systemic diseases**					
**Hyperthyroidism**	3.96 (2.09–7.48)	1.82	0.78–4.24	0.163	0.383
**Hypothyroidism**	1.59 (0.99–2.55)	0.85	0.45–1.59	0.609	0.807
**Rheumatoid arthritis**	2.17 (1.33–3.55)	1.03	0.53–2.03	0.920	0.933
**Allergic rhinitis or sinusitis**	**2.26 (1.75–2.91)**	**1.86**	**1.38–2.52**	**<0.001**	**0.002**
**Autoimmune diseases**	2.49 (1.28–4.83)	0.49	0.17–1.43	0.193	0.429
**Asthma**	2.21 (1.57–3.09)	1.28	0.85–1.92	0.233	0.466
**Hypercholesterolaemia**	1.46 (1.06–2.03)	0.89	0.57–1.40	0.625	0.807
**Diabetes mellitus**	1.76 (1.17–2.63)	1.25	0.73–2.12	0.415	0.615
**Hypertension**	2.33 (1.58–3.44)	1.74	1.04–2.91	0.035	0.157
**Depression**	2.00 (1.40–2.85)	0.82	0.51–1.32	0.408	0.615
**Chronic anxiety disorder**	2.53 (1.82–3.53)	1.67	1.09–2.55	0.018	0.098
**Physical activity (reference: No weekly physical activity)**					
**<30 min moderate/vigorous activity, 5 days/week**	0.71 (0.56–0.89)	0.74	0.57–0.95	0.017	0.098
**≥30 min moderate/vigorous activity, 5 days/week**	**0.54 (0.39–0.74)**	**0.57**	**0.41–0.81**	**0.002**	**0.019**

*Abbreviations: aOR: adjusted odds ratio; CI: confidence interval; BMI: body mass index; SAR: Saudi Arabian Riyal. Bold rows indicate statistically significant associations (p < 0.05).*

## Data Availability

The original survey exports, the de-identified analysis dataset, and the scoring dictionary for the primary outcome are available on request from the corresponding author, and can be supplied on request. The data are not publicly available due to ethical restrictions and privacy concerns related to sensitive health information.

## Supplementary Materials

The following supporting information can be downloaded at: https://www.mdpi.com/article/10.3390/healthcare14193150/s1, File S1: Study questionnaire as administered (Arabic and English versions); Table S1: Reduced model excluding covariates with fewer than 20 exposed participants; Table S2: Model with previously diagnosed ocular surface and eyelid margin conditions added; Table S3: Model restricted to participants without any ocular surface or eyelid margin diagnosis; Table S4: Model with the outcome restricted to recurrence of at least several times per month during the preceding six months; Table S5: Firth penalized maximum likelihood model with profile penalized likelihood confidence intervals; Table S6: Model reinstating the records excluded for implausible anthropometry, with body mass index omitted; Table S7: Calibration of the primary model by decile of predicted probability.

## Abbreviations

The following abbreviations are used in this manuscript:

AI	Artificial Intelligence
aOR	Adjusted Odds Ratio
BMI	Body Mass Index
CI	Confidence Interval
R^2^	Coefficient of Determination
SAR	Saudi Arabian Riyal
SD	Standard Deviation
STROBE	Strengthening the Reporting of Observational Studies in Epidemiology

## References

[1] Cronau H., Kankanala R.R., Mauger T. (2010). Diagnosis and Management of Red Eye in Primary Care. Am. Fam. Physician.

[2] Shalini H., Bali A., Poojitha S. (2025). Knowledge, Attitude, and Practices Toward Conjunctivitis Among General Population of Central Karnataka: A Cross-Sectional Study. J. Fam. Med. Prim. Care.

[3] Leonardi A., Castegnaro A., Valerio A.L., Lazzarini D. (2015). Epidemiology of Allergic Conjunctivitis: Clinical Appearance and Treatment Patterns in a Population-Based Study. Curr. Opin. Allergy Clin. Immunol..

[4] Singh K., Axelrod S., Bielory L. (2010). The Epidemiology of Ocular and Nasal Allergy in the United States, 1988–1994. J. Allergy Clin. Immunol..

[5] Bielory L., Delgado L., Katelaris C.H., Leonardi A., Rosario N., Vichyanoud P. (2020). ICON: Diagnosis and Management of Allergic Conjunctivitis. Ann. Allergy Asthma Immunol..

[6] Villegas B.V., Benitez-Del-Castillo J.M. (2021). Current Knowledge in Allergic Conjunctivitis. Turk. J. Ophthalmol..

[7] Britten-Jones A.C., Wang M.T.M., Samuels I., Jennings C., Stapleton F., Craig J.P. (2024). Epidemiology and Risk Factors of Dry Eye Disease: Considerations for Clinical Management. Medicina.

[8] Bashir M., Alghamdi T.A., Alzahrani A.M., Alghamdi M.A.A., Aloleeit S.K., AlHajji Z., Alsultan E.S., Aljamaan N.H., Abu Rukbah L.K. (2024). Evaluation of Allergic Conjunctivitis Prevalence and Attitude Toward Prevention and Control in Saudi Arabia. Cureus.

[9] Al Hadlaq A.M., Al Harbi M.M. (2023). The Epidemiological Profile and Predisposing Factors of Microbial Keratitis Over a 10-Year Period at a Tertiary Hospital in Central Saudi Arabia. Cureus.

[10] Hosten L.O., Snyder C. (2020). Over-the-Counter Ocular Decongestants in the United States: Mechanisms of Action and Clinical Utility for Management of Ocular Redness. Clin. Optom..

[11] Soparkar C.N., Wilhelmus K.R., Koch D.D., Wallace G.W., Jones D.B. (1997). Acute and Chronic Conjunctivitis Due to Over-the-Counter Ophthalmic Decongestants. Arch. Ophthalmol..

[12] Bousquet J., Van Cauwenberge P., Khaltaev N. (2001). Allergic Rhinitis and Its Impact on Asthma. J. Allergy Clin. Immunol..

[13] Sánchez-Hernández M.C., Navarro A.M., Colás C., Del Cuvillo A., Sastre J., Mullol J., Valero A. (2020). Validation of the DECA Criteria for Allergic Conjunctivitis Severity and Control. Clin. Transl. Allergy.

[14] Frings A., Geerling G., Schargus M. (2017). Red Eye: A Guide for Non-Specialists. Dtsch. Arztebl. Int..

[15] Short M.E., Goetzel R.Z., Pei X., Tabrizi M.J., Ozminkowski R.J., Gibson T.B., DeJoy D.M., Wilson M.G. (2009). How Accurate Are Self-Reports? Analysis of Self-Reported Health Care Utilization and Absence When Compared with Administrative Data. J. Occup. Environ. Med..

[16] Al-Mohtaseb Z., Schachter S., Shen Lee B., Garlich J., Trattler W. (2021). The Relationship Between Dry Eye Disease and Digital Screen Use. Clin. Ophthalmol..

[17] Albalawi E.D., Alswayed S.K., Aldharman S.S., Alshangiti A.Y., Alhussein G.A., Alamawi H.O. (2023). The Association of Screen Time, Sleep Quality, and Dry Eye Among College Students in Saudi Arabia. Cureus.

[18] Allergic Conjunctivitis. https://www.ncbi.nlm.nih.gov/books/NBK448118/.

[19] Al Hemaidi S., Alharthi A., AlFehaid M., Almalki H., Hamzi Y.A., Alnemari A., Alhajri N.M., Al Hunaif A.M., Almaghrebi D.A., Alabdulminaim J. (2025). Knowledge, Attitude, and Practice Regarding Over-the-Counter Prescription of Topical Eye Medications Among the General Population of Saudi Arabia. Cureus.

[20] Sheppard J.D., Nichols K.K. (2023). Dry Eye Disease Associated with Meibomian Gland Dysfunction: Focus on Tear Film Characteristics and the Therapeutic Landscape. Ophthalmol. Ther..

[21] Wang M.T., Craig J.P. (2018). Investigating the Effect of Eye Cosmetics on the Tear Film: Current Insights. Clin. Optom..

[22] Ng A., Evans K., North R., Purslow C. (2012). Eye Cosmetic Usage and Associated Ocular Comfort. Ophthalmic Physiol. Opt..

[23] Perkin M.R., Bader T., Rudnicka A.R., Strachan D.P., Owen C.G. (2015). Inter-Relationship Between Rhinitis and Conjunctivitis in Allergic Rhinoconjunctivitis and Associated Risk Factors in Rural UK Children. PLoS ONE.

[24] Bielory L., Ghafoor S. (2005). Histamine Receptors and the Conjunctiva. Curr. Opin. Allergy Clin. Immunol..

[25] Qian L., Wei W. (2022). Identified Risk Factors for Dry Eye Syndrome: A Systematic Review and Meta-Analysis. PLoS ONE.

[26] Lin Y.L., Liu H.H., Chen S.J., Wan Q.H., Zhang K.P. (2026). Correlation of Ocular Surface Function with Sleep Quality, Anxiety, and Depression in Patients with Dry Eye Disease. World J. Psychiatry.

[27] Teixeira R.R., Díaz M.M., Santos T.V., Bernardes J.T., Peixoto L.G., Bocanegra O.L., Neto M.B., Espindola F.S. (2015). Chronic Stress Induces a Hyporeactivity of the Autonomic Nervous System in Response to Acute Mental Stressor and Impairs Cognitive Performance in Business Executives. PLoS ONE.

[28] Zhang Q., Jiang Y., Deng C., Wang J. (2024). Effects and Potential Mechanisms of Exercise and Physical Activity on Eye Health and Ocular Diseases. Front. Med..

[29] Borrelli M., Frings A., Geerling G., Finis D. (2021). Gender-Specific Differences in Signs and Symptoms of Dry Eye Disease. Curr. Eye Res..

