# Supplementary material for: Self-Reported Red Eye Among Saudi Adults: Symptom Profile, Self-Management Practices, and Associated Factors
Source: Healthcare (Basel). 2026 Sep 22;14(19):3150. doi: 10.3390/healthcare14193150 (PMC13665425; doi:10.3390/healthcare14193150)
Supplement: Supplementary file 1 [file healthcare-14-03150-s001.zip › File S1-Red Eye and Its Treatment Methods.pdf]

Red Eye and Its Treatment Methods

🎯 We are a research group from several Saudi universities, and we invite you to participate in a scientific study entitled **“Red Eye and Its Treatment Methods”** by completing this questionnaire as outlined below:

🔐 The information you provide will remain completely confidential, with full protection of your privacy, and will be used solely for research and scientific purposes.

🔎 The study consists of four sections:

- Sociodemographic information
- Eye-related medical history
- Dealing with the appearance of the eye
- A self-esteem assessment questionnaire

✍️ Completing the questionnaire will take no more than 5 minutes of your time.

📢 Participation is voluntary, and you may

withdraw from the study at any time, even after providing consent to participate.

If you have any questions or wish to withdraw, please contact:
sajaalasiri1@gmail.com
dhiyaaot@gmail.com

* Indicates required question

1. **Your completion of the questionnaire indicates your consent to participate in the study.** *

Mark only one oval.

- ( ) Yes

2. **Data Collector Number** * Dropdown

Mark only one oval.

- ( ) 1
- ( ) 2
- ( ) 3

Demographic Information

This information helps us examine the relationship between participants' characteristics and their level of self-esteem.

3. **Gender** *

Mark only one oval.

- [ ] Male
- [ ] Female

4. **Nationality** *

Mark only one oval.

- [ ] Saudi
- [ ] Non-Saudi

5. **Age** *

____

6. **Weight in Kg** *
Example: 60

____

7. **Height in cm** *
Example: 160

____

8. **Region** *

Dropdown

Mark only one oval.

- Southern Region
- Eastern Region
- Northern Region
- Western Region
- Central Region

9. **Marital Status** *

Mark only one oval.

- Single
- Married
- Divorced or Widowed

10. **Place of Residence** *

Mark only one oval.

- City
- Village

11. **Level of Education** *

Mark only one oval.

- Secondary school or below
- University (Bachelor's degree or Diploma)
- Postgraduate studies (Master's or Doctorate)

12. **If you are employed, what is your work sector?** *

Mark only one oval.

- Student
- Unemployed
- Healthcare sector
- Education sector
- Military sector
- Administrative sector
- Agricultural sector
- Industrial sector
- Handicrafts and self-employed work (such as carpentry, plumbing, or blacksmithing)

13. **Monthly Household Income** *

Mark only one oval.

- Less than 5,000 Saudi Riyals
- 5,000–9,999 Saudi Riyals
- 10,000–14,999 Saudi Riyals
- More than 15,000 Saudi Riyals

14. **Have you been diagnosed with any of the following conditions?** *

Mark only one oval per row.

	Yes	No
**Allergic conjunctivitis (pink eye)**		
**Chronic dry eye**		
**Keratitis**		
**Chronic blepharitis**		
**Atopic dermatitis (eczema) of the eyelids**		
**Redness and bumps around the eyes**		
**Facial nerve palsy (Bell's palsy)**		
**Hyperthyroidism**		
**Hypothyroidism**		
**Rheumatoid arthritis**		

**Allergic rhinitis and sinusitis**	⬭	⬭
**Glaucoma**	⬭	⬭
**Autoimmune diseases (e.g., systemic lupus erythematosus, Sjögren's syndrome)**	⬭	⬭
**Asthma**	⬭	⬭
**Hyperlipidemia / high cholesterol**	⬭	⬭
**Diabetes mellitus**	⬭	⬭
**Hypertension**	⬭	⬭
**Depression**	⬭	⬭
**Chronic / excessive anxiety**	⬭	⬭

15. **Use:** *

Mark only one oval per row.

	Yes	No
**Do you use prescription eyeglasses?**	⬭	⬭
**Do you use soft contact lenses (medical or cosmetic)?**	⬭	⬭
**Do you use rigid contact lenses?**	⬭	⬭
**Do you use scleral contact lenses?**	⬭	⬭
**Cosmetics**	⬭	⬭

16. **Surgical Procedures:** *

Mark only one oval per row.

	Yes	No
**Refractive (vision correction) surgery**	⬭	⬭
**Cataract surgery**	⬭	⬭
**Glaucoma surgery**	⬭	⬭
**Strabismus (squint) correction surgery**	⬭	⬭

17. **Level of Physical Activity:** *

Mark only one oval.

- I do not engage in any physical activity during the week.
- I engage in moderate or vigorous physical activity for less than 30 minutes, 5 times per week.
- I engage in moderate or vigorous physical activity for at least 30 minutes, 5 times per week.

18. **Are you a smoker?** *

Smoking is not limited to cigarette use; it also includes <u>shisha, hookah, electronic cigarettes (vaping), and pipe smoking.</u>

Mark only one oval.

- Yes
- No, I have never smoked
- Ex-smoker

19. **Do you currently have, or have you previously had, red eye?** *

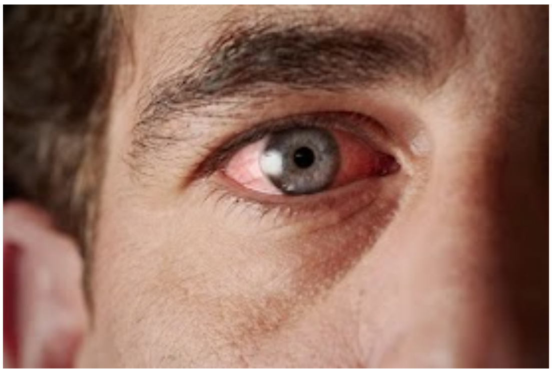

Mark only one oval.

- Yes
- No *Skip to question 37*

Red Eye

20. **Did red eye negatively affect your daily life and interaction with others (such as at work, university, or with family and friends)?** *

Mark only one oval.

- Did not affect
- Affected slightly
- Affected moderately
- Affected severely

21. **How often have you experienced red eye during the past six months?** *

Mark only one oval.

- Daily or almost daily
- Several times per week
- Several times per month
- Rarely (less than once per month)

22. **Usually, how long does a red eye episode last?** *

Mark only one oval.

- Less than 48 hours
- Less than one week
- One week to four weeks
- More than four weeks

23. **Have you experienced any of the following associated symptoms?** *

Mark only one oval per row.

	Yes	No
**Itching**	◯	◯
**Burning or stinging sensation**	◯	◯
**Foreign body sensation (gritty or sandy feeling)**	◯	◯
**Discharge (sticky or purulent)**	◯	◯
**Increased tearing**	◯	◯
**Pain**	◯	◯
**Blurred vision**	◯	◯
**Light sensitivity (photophobia)**	◯	◯

24. **Which of the following factors cause or worsen red eye?** *

Mark only one oval per row.

	Yes	No
**Prolonged use of smart device screens**	⬭	⬭
**Lack of sleep**	⬭	⬭
**Allergy**	⬭	⬭
**Contact lens use**	⬭	⬭
**Exposure to smoke or fumes**	⬭	⬭
**During or after swimming**	⬭	⬭
**After rubbing the eyes**	⬭	⬭

25. **Did you need to visit a doctor to treat red eye?** *

Mark only one oval.

- ⬭ Yes
- ⬭ No

Red eye managment choices

26. **What treatment methods do you use to manage red eye?** *

Mark only one oval per row.

	Yes	No
**Nothing; I wait until the symptoms resolve**	◯	◯
**Washing the eyes with water**	◯	◯
**Using warm or cold compresses**	◯	◯
**Using lubricating eye drops**	◯	◯
**Using allergy medications**	◯	◯
**Using antibiotic eye drops**	◯	◯
**Using steroid anti-inflammatory eye drops**	◯	◯

27. **On a scale from 0 to 10, how would you rate the improvement in your red eye symptoms after using these treatment methods?** *

Mark only one oval.

	No improvement
0	⬭
1	⬭
2	⬭
3	⬭
4	⬭
5	⬭
6	⬭
7	⬭
8	⬭
9	⬭
10	⬭
	Very marked improvement

Use of eye-whitening drops

28. **Have you ever used eye-whitening drops?** *

Mark only one oval.

- Yes *Skip to question 29*
- No *Skip to question 37*

Characteristics and Complications of Eye-Whitening Drops

29. **When did you start using eye-whitening drops?** *

Mark only one oval.

- Less than three months ago
- Three to six months ago
- More than six months ago

30. **How often have you used eye-whitening drops during the past six months?** *

____

31. **Did you use eye-whitening drops based on a physician’s recommendation?** *

Mark only one oval.

- Yes
- No

32. **Which eye-whitening drop did you use?** *

Mark only one oval.

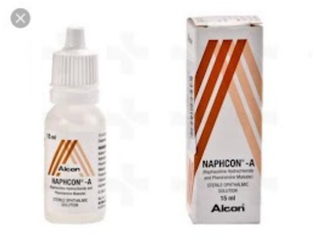

◯ Naphcon-A

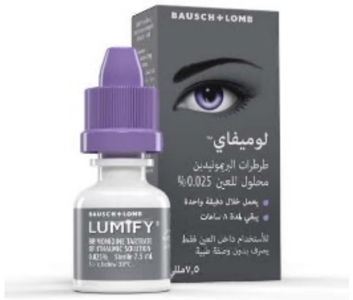

◯ Lumify

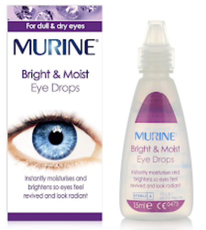

◯ Murine (Bright & Moist)

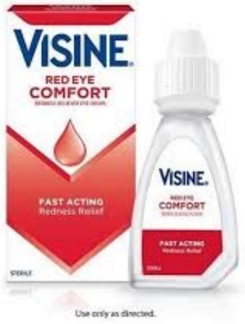

◯ Visine

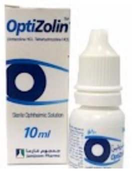

◯ Optizoline

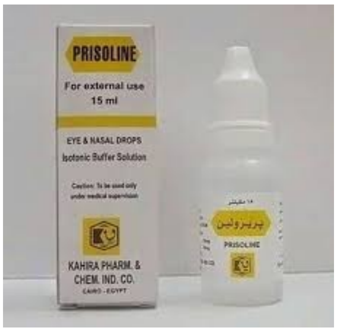

◯ Brizoline

33. **What complications did you notice after using eye-whitening drops?** *

Mark only one oval per row.

	Yes	No
**Burning or stinging sensation in the eye**	⬭	⬭
**Foreign body sensation**	⬭	⬭
**Increased tearing**	⬭	⬭
**Blurred vision for several minutes**	⬭	⬭
**Headache**	⬭	⬭
**Palpitations (rapid heartbeat)**	⬭	⬭

34. **With repeated use of eye-whitening drops for weeks or months, did you notice any of the following?** *

Mark only one oval per row.

	Yes	No
**Redness worsens when I stop using the drops**	⬭	⬭
**I need to use the drops more frequently to achieve the same effect**	⬭	⬭
**Increased eye dryness or irritation**	⬭	⬭
**Eyelid or skin sensitivity (redness, itching, eczema)**	⬭	⬭

35. **On a scale from 0 to 10, how much did your red eye symptoms improve after using eye-whitening drops?** *

Mark only one oval.

	No improvement
0	◯
1	◯
2	◯
3	◯
4	◯
5	◯
6	◯
7	◯
8	◯
9	◯
10	◯
	Very marked improvement

36. **On a scale from 0 to 10, how much discomfort or overall problems did eye-whitening drops cause you?** *

Mark only one oval.

	No problems at all
0	⬭
1	⬭
2	⬭
3	⬭
4	⬭
5	⬭
6	⬭
7	⬭
8	⬭
9	⬭
10	⬭
	Caused major problems

Rosenberg Self-Esteem Scale

Please record the appropriate answer for each item, depending on whether you **Strongly agree, agree, disagree, or strongly disagree with it.**

37. **Self-Esteem Questionnaire – Rosenberg Self-Esteem Scale** *

Please use your phone in **landscape mode** to view the response options more clearly.

Mark only one oval per row.

	Strongly disagree	Disagree	Agree	Strongly agree
**On the whole, I am satisfied with myself.**	⬭	⬭	⬭	⬭
**At times I think I am no good at all.**	⬭	⬭	⬭	⬭
**I feel that I have a number of good qualities.**	⬭	⬭	⬭	⬭
**I am able to do things as well as most**	⬭	⬭	⬭	⬭

other people.				
I feel I do not have much to be proud of.	◯	◯	◯	◯
I certainly feel useless at times.	◯	◯	◯	◯
I feel that I'm a person of worth.	◯	◯	◯	◯
I wish I could have more respect for myself.	◯	◯	◯	◯
All in all, I am inclined to think that I am a failure.	◯	◯	◯	◯
I take a positive attitude toward myself.	◯	◯	◯	◯

Thank you for your contribution to this research initiative ✨
Your participation helps advance and enrich scientific research in the region.

This content is neither created nor endorsed by Google.

Google Forms
