# Supplementary material for: Self-Reported Red Eye Among Saudi Adults: Symptom Profile, Self-Management Practices, and Associated Factors
Source: Healthcare (Basel). 2026 Sep 22;14(19):3150. doi: 10.3390/healthcare14193150 (PMC13665425; doi:10.3390/healthcare14193150)
Supplement: Supplementary file 1 [file healthcare-14-03150-s001.zip › healthcare-4554649-supplementary.pdf]

Supplementary Material

The robustness of the primary model was examined using Firth penalized estimation and five sensitivity analyses.

Sparse covariates. Excluding the eight covariates with fewer than 20 exposed participants raised the ratio to 14.8 events per parameter and left the principal estimates unchanged (Table S1). Firth penalized maximum likelihood, fitted with profile penalized likelihood confidence intervals, contracted the intervals of the sparse ocular surgery terms: glaucoma surgery moved from 3.67 (95% CI: 0.49–27.60) to 2.62 (95% CI: 0.45–18.87), and cataract surgery from 0.16 (0.02–1.49) to 0.22 (0.02–1.39). Estimates for male sex (1.72), cosmetic use (1.72), allergic rhinitis or sinusitis (1.84), hypertension (1.69), chronic anxiety disorder (1.63), and the highest physical activity category (0.59) were materially unchanged, indicating that the principal findings are not artefacts of sparse-data bias (Table S5).

Pre-existing ocular surface disease. Adding previously diagnosed ocular surface and eyelid margin conditions to the model strengthened the association with cosmetic use (aOR = 2.02; 95% CI: 1.36–3.00; $p = 0.001$) and raised Tjur's R^2 to 0.164 (Table S2). Restricting the sample to the 1,377 participants without any such diagnosis, among whom 333 (24.2%) reported red eye, preserved both cosmetic use (aOR = 2.04; 95% CI: 1.28–3.24; $p = 0.003$) and physical activity (aOR = 0.51; 95% CI: 0.35–0.77; $p = 0.001$) (Table S3). The lifestyle associations are therefore not explained by pre-existing ocular surface disease.

Recent-onset outcome. Restricting the outcome to the 171 participants reporting recurrence at least several times per month during the preceding six months, so that outcome and exposures refer to a common recent period, preserved the associations with allergic rhinitis or sinusitis (aOR = 1.88; 95% CI: 1.20–2.95), cosmetic use (aOR = 1.88; 95% CI: 1.06–3.36), and a bachelor's degree or diploma (aOR = 1.79; 95% CI: 1.12–2.86), and strengthened that with hypertension (aOR = 2.89; 95% CI: 1.42–5.86). Male sex (aOR = 1.56; 95% CI: 0.89–2.73), chronic anxiety disorder (aOR = 1.55; 95% CI: 0.82–2.91), and physical activity (aOR = 0.72; 95% CI: 0.42–1.23) attenuated towards the null (Table S4). Only 31 participants reported episodes lasting one week or longer, too few to support a stable model.

Anthropometric exclusions. Reinstating the 26 records excluded for implausible anthropometry, with body mass index omitted, gave 1,653 participants and 477 affected (28.9%) and left every estimate essentially unchanged, for example cosmetic use 1.75 against 1.74 and allergic rhinitis or sinusitis 1.89 against 1.86 (Table S6). Body mass index was not associated with the outcome in the primary model (aOR = 1.02; 95% CI: 1.00–1.04; $p = 0.127$), so its handling does not affect the reported associations

Table S1. Reduced model excluding covariates with fewer than 20 exposed participants (474 cases; n = 1,627).

Variable	Adjusted OR	95% CI	p-value
Male sex	1.76	1.24 to 2.51	0.002
Saudi nationality	0.75	0.54 to 1.03	0.080
Married	1.01	0.67 to 1.52	0.952
Divorced or widowed	0.91	0.41 to 2.00	0.814
Urban residence	0.92	0.65 to 1.30	0.619
Bachelor's degree or diploma	1.70	1.27 to 2.27	<0.001
Postgraduate studies	1.92	1.12 to 3.30	0.019
Employed	1.00	0.67 to 1.47	0.982
Student	1.41	0.93 to 2.13	0.104
Income 5,000–9,999 SAR	1.04	0.74 to 1.45	0.842
Income 10,000–14,999 SAR	0.81	0.57 to 1.15	0.247
Income >15,000 SAR	1.16	0.85 to 1.58	0.355
Current smoker	1.12	0.78 to 1.63	0.537
Ex-smoker	1.10	0.72 to 1.70	0.653
Physical activity <30 min, 5 days/week	0.75	0.58 to 0.96	0.025
Physical activity ≥30 min, 5 days/week	0.57	0.40 to 0.81	0.001
Age (years)	0.99	0.97 to 1.00	0.087
BMI (kg/m²)	1.02	1.00 to 1.04	0.122
Eyeglasses	1.27	0.99 to 1.62	0.062
Soft contact lenses	1.40	0.97 to 2.00	0.069
Cosmetic use around the eyes	1.75	1.21 to 2.53	0.003
Refractive surgery	0.95	0.63 to 1.44	0.810
Hyperthyroidism	1.74	0.79 to 3.82	0.166
Hypothyroidism	0.90	0.50 to 1.65	0.741
Rheumatoid arthritis	1.10	0.58 to 2.09	0.763
Allergic rhinitis or sinusitis	1.87	1.39 to 2.53	<0.001
Asthma	1.28	0.86 to 1.91	0.217
Hypercholesterolemia	0.90	0.58 to 1.39	0.627
Diabetes mellitus	1.29	0.77 to 2.17	0.339
Hypertension	1.71	1.03 to 2.84	0.039
Depression	0.85	0.53 to 1.35	0.484
Chronic anxiety disorder	1.63	1.07 to 2.49	0.022

Table S2. Model with previously diagnosed ocular surface and eyelid margin conditions added (474 cases; n = 1,627).

Variable	Adjusted OR	95% CI	p-value
Male sex	1.99	1.36 to 2.91	<0.001
Saudi nationality	0.85	0.60 to 1.19	0.343
Married	1.00	0.65 to 1.54	0.985
Divorced or widowed	1.03	0.45 to 2.39	0.938
Urban residence	0.90	0.63 to 1.29	0.569
Bachelor's degree or diploma	1.65	1.23 to 2.23	<0.001
Postgraduate studies	1.83	1.03 to 3.23	0.038
Employed	0.97	0.64 to 1.46	0.880
Student	1.44	0.94 to 2.21	0.097
Income 5,000–9,999 SAR	0.96	0.67 to 1.37	0.830
Income 10,000–14,999 SAR	0.80	0.56 to 1.15	0.220
Income >15,000 SAR	1.14	0.82 to 1.57	0.434
Current smoker	1.20	0.81 to 1.76	0.367
Ex-smoker	1.20	0.77 to 1.87	0.431
Physical activity <30 min, 5 days/week	0.75	0.57 to 0.97	0.030
Physical activity ≥30 min, 5 days/week	0.58	0.41 to 0.83	0.003
Age (years)	0.98	0.97 to 1.00	0.061
BMI (kg/m²)	1.02	1.00 to 1.04	0.052
Eyeglasses	1.11	0.85 to 1.44	0.454
Soft contact lenses	1.26	0.85 to 1.86	0.252
Rigid contact lenses	1.49	0.59 to 3.75	0.395
Scleral contact lenses	1.11	0.46 to 2.66	0.818
Cosmetic use around the eyes	2.02	1.36 to 3.00	<0.001
Refractive surgery	0.90	0.58 to 1.40	0.628
Cataract surgery	0.11	0.01 to 1.33	0.083
Glaucoma surgery	5.81	0.71 to 47.90	0.102
Strabismus surgery	1.76	0.51 to 6.12	0.373
Glaucoma	0.35	0.07 to 1.79	0.208
Facial nerve palsy	0.82	0.19 to 3.56	0.788
Hyperthyroidism	1.71	0.70 to 4.16	0.237
Hypothyroidism	0.87	0.45 to 1.69	0.676
Rheumatoid arthritis	0.85	0.40 to 1.80	0.667
Allergic rhinitis or sinusitis	1.68	1.22 to 2.31	0.001
Autoimmune diseases	0.29	0.08 to 1.13	0.075
Asthma	1.34	0.88 to 2.04	0.180
Hypercholesterolemia	0.68	0.42 to 1.11	0.120
Diabetes mellitus	1.35	0.77 to 2.36	0.290
Hypertension	1.72	0.98 to 3.00	0.057
Depression	0.79	0.48 to 1.31	0.359
Chronic anxiety disorder	1.40	0.88 to 2.21	0.154
Chronic dry eye disease	2.98	1.92 to 4.62	<0.001
Allergic conjunctivitis	12.51	5.19 to 30.19	<0.001
Chronic blepharitis	1.57	0.50 to 4.90	0.438
Keratitis	1.27	0.51 to 3.16	0.610
Atopic dermatitis of the eyelids	0.19	0.06 to 0.58	0.003
Periocular redness or inflammation	2.02	1.07 to 3.80	0.030

Table S3. Model restricted to participants without any ocular surface or eyelid margin diagnosis (333 cases; n = 1,377).

Variable	Adjusted OR	95% CI	p-value
Male sex	1.83	1.19 to 2.81	0.006
Saudi nationality	0.88	0.60 to 1.30	0.519
Married	0.86	0.53 to 1.40	0.540
Divorced or widowed	0.89	0.35 to 2.27	0.802
Urban residence	0.86	0.58 to 1.27	0.444
Bachelor's degree or diploma	1.56	1.12 to 2.16	0.008
Postgraduate studies	1.68	0.87 to 3.28	0.125
Employed	0.96	0.60 to 1.52	0.850
Student	1.25	0.78 to 2.01	0.345
Income 5,000–9,999 SAR	0.83	0.56 to 1.24	0.361
Income 10,000–14,999 SAR	0.73	0.49 to 1.08	0.118
Income >15,000 SAR	1.08	0.76 to 1.53	0.662
Current smoker	1.12	0.72 to 1.74	0.617
Ex-smoker	1.42	0.88 to 2.31	0.151
Physical activity <30 min, 5 days/week	0.63	0.47 to 0.84	0.002
Physical activity ≥30 min, 5 days/week	0.51	0.35 to 0.77	0.001
Age (years)	0.98	0.96 to 1.00	0.060
BMI (kg/m²)	1.03	1.00 to 1.05	0.025
Eyeglasses	1.12	0.84 to 1.50	0.446
Soft contact lenses	1.33	0.85 to 2.08	0.208
Rigid contact lenses	1.34	0.42 to 4.28	0.618
Scleral contact lenses	0.68	0.18 to 2.51	0.563
Cosmetic use around the eyes	2.04	1.28 to 3.24	0.003
Refractive surgery	0.76	0.45 to 1.27	0.290
Cataract surgery	—	not estimable	—
Glaucoma surgery	1.67	0.06 to 47.71	0.765
Strabismus surgery	5.35	1.06 to 27.04	0.043
Glaucoma	1.96	0.07 to 55.85	0.694
Facial nerve palsy	5.97	0.74 to 48.13	0.093
Hyperthyroidism	1.82	0.59 to 5.64	0.298
Hypothyroidism	0.86	0.38 to 1.90	0.703
Rheumatoid arthritis	0.76	0.25 to 2.27	0.618
Allergic rhinitis or sinusitis	1.91	1.31 to 2.77	<0.001
Autoimmune diseases	0.62	0.13 to 2.92	0.545
Asthma	1.43	0.87 to 2.36	0.161
Hypercholesterolemia	0.69	0.37 to 1.29	0.250
Diabetes mellitus	0.90	0.43 to 1.88	0.785
Hypertension	1.90	0.94 to 3.85	0.074
Depression	0.74	0.41 to 1.33	0.316
Chronic anxiety disorder	1.48	0.85 to 2.57	0.171

Cataract surgery could not be estimated in this subsample because no participant with that history reported the outcome (complete separation).

Table S4. Model with the outcome restricted to recurrence of at least several times per month during the preceding six months (171 cases; n = 1,324).

Variable	Adjusted OR	95% CI	p-value
Male sex	1.56	0.89 to 2.73	0.118
Saudi nationality	0.92	0.56 to 1.51	0.754
Married	1.29	0.68 to 2.46	0.441
Divorced or widowed	0.54	0.12 to 2.44	0.426
Urban residence	1.48	0.79 to 2.79	0.220
Bachelor's degree or diploma	1.79	1.12 to 2.86	0.015
Postgraduate studies	2.17	0.94 to 5.02	0.071
Employed	1.30	0.68 to 2.46	0.426
Student	2.62	1.31 to 5.24	0.007
Income 5,000–9,999 SAR	1.35	0.81 to 2.25	0.256
Income 10,000–14,999 SAR	0.89	0.51 to 1.53	0.662
Income >15,000 SAR	0.91	0.55 to 1.49	0.694
Current smoker	1.76	1.05 to 2.96	0.032
Ex-smoker	1.33	0.68 to 2.59	0.406
Physical activity <30 min, 5 days/week	0.96	0.65 to 1.44	0.860
Physical activity ≥30 min, 5 days/week	0.72	0.42 to 1.23	0.233
Age (years)	1.00	0.97 to 1.03	0.988
BMI (kg/m²)	0.98	0.94 to 1.02	0.251
Eyeglasses	1.44	0.98 to 2.11	0.060
Soft contact lenses	1.47	0.86 to 2.53	0.162
Rigid contact lenses	0.56	0.16 to 1.94	0.360
Scleral contact lenses	3.13	1.15 to 8.55	0.026
Cosmetic use around the eyes	1.88	1.06 to 3.36	0.031
Refractive surgery	0.90	0.46 to 1.78	0.769
Cataract surgery	0.14	0.01 to 2.06	0.153
Glaucoma surgery	3.49	0.27 to 45.73	0.340
Strabismus surgery	2.39	0.53 to 10.78	0.256
Glaucoma	1.87	0.30 to 11.56	0.499
Facial nerve palsy	4.54	0.92 to 22.33	0.063
Hyperthyroidism	1.29	0.36 to 4.56	0.698
Hypothyroidism	1.07	0.45 to 2.56	0.884
Rheumatoid arthritis	0.91	0.34 to 2.48	0.860
Allergic rhinitis or sinusitis	1.88	1.20 to 2.95	0.006
Autoimmune diseases	0.40	0.08 to 2.02	0.270
Asthma	1.59	0.90 to 2.79	0.109
Hypercholesterolemia	0.79	0.41 to 1.55	0.496
Diabetes mellitus	0.90	0.40 to 2.02	0.800
Hypertension	2.89	1.42 to 5.86	0.003
Depression	0.84	0.41 to 1.71	0.624
Chronic anxiety disorder	1.55	0.82 to 2.91	0.176

Table S5. Firth penalized maximum likelihood model with profile penalized likelihood confidence intervals (474 cases; n = 1,627).

Variable	Penalized OR	95% CI	p-value
Male sex	1.72	1.22 to 2.46	0.002
Saudi nationality	0.75	0.55 to 1.03	0.078
Married	1.03	0.69 to 1.54	0.893
Divorced or widowed	0.98	0.44 to 2.08	0.967
Urban residence	0.91	0.65 to 1.29	0.582
Bachelor's degree or diploma	1.68	1.27 to 2.25	<0.001
Postgraduate studies	1.89	1.11 to 3.22	0.020
Employed	0.98	0.67 to 1.45	0.934
Student	1.40	0.93 to 2.11	0.104
Income 5,000–9,999 SAR	1.05	0.75 to 1.47	0.773
Income 10,000–14,999 SAR	0.83	0.59 to 1.17	0.291
Income >15,000 SAR	1.16	0.85 to 1.58	0.343
Current smoker	1.12	0.77 to 1.61	0.552
Ex-smoker	1.11	0.72 to 1.68	0.647
Physical activity <30 min, 5 days/week	0.74	0.58 to 0.95	0.018
Physical activity ≥30 min, 5 days/week	0.59	0.41 to 0.82	0.002
Age (years)	0.99	0.97 to 1.00	0.126
BMI (kg/m²)	1.02	1.00 to 1.04	0.130
Eyeglasses	1.25	0.98 to 1.60	0.077
Soft contact lenses	1.30	0.90 to 1.88	0.156
Rigid contact lenses	1.12	0.47 to 2.62	0.790
Scleral contact lenses	1.48	0.65 to 3.30	0.345
Cosmetic use around the eyes	1.72	1.19 to 2.48	0.004
Refractive surgery	0.95	0.62 to 1.43	0.797
Cataract surgery	0.22	0.02 to 1.39	0.113
Glaucoma surgery	2.62	0.45 to 18.87	0.286
Strabismus surgery	1.69	0.53 to 5.32	0.367
Glaucoma	1.08	0.28 to 3.86	0.909
Facial nerve palsy	1.70	0.48 to 5.79	0.403
Hyperthyroidism	1.72	0.76 to 3.89	0.193
Hypothyroidism	0.87	0.46 to 1.57	0.640
Rheumatoid arthritis	1.03	0.52 to 1.95	0.940
Allergic rhinitis or sinusitis	1.84	1.36 to 2.47	<0.001
Autoimmune diseases	0.52	0.18 to 1.37	0.194
Asthma	1.27	0.85 to 1.88	0.248
Hypercholesterolemia	0.90	0.58 to 1.39	0.653
Diabetes mellitus	1.24	0.74 to 2.07	0.411
Hypertension	1.69	1.02 to 2.78	0.043
Depression	0.83	0.52 to 1.32	0.438
Chronic anxiety disorder	1.63	1.07 to 2.48	0.022

Table S6. Model reinstating the 26 records excluded for implausible anthropometry, with body mass index omitted (477 cases; n = 1,653).

Variable	Adjusted OR	95% CI	p-value
Male sex	1.82	1.28 to 2.58	<0.001
Saudi nationality	0.76	0.55 to 1.04	0.091
Married	1.10	0.73 to 1.64	0.648
Divorced or widowed	0.98	0.45 to 2.12	0.963
Urban residence	0.92	0.65 to 1.30	0.626
Bachelor's degree or diploma	1.73	1.30 to 2.31	<0.001
Postgraduate studies	1.94	1.13 to 3.33	0.016
Employed	0.96	0.65 to 1.42	0.845
Student	1.41	0.93 to 2.13	0.101
Income 5,000–9,999 SAR	1.05	0.74 to 1.47	0.795
Income 10,000–14,999 SAR	0.83	0.58 to 1.17	0.290
Income >15,000 SAR	1.17	0.86 to 1.60	0.313
Current smoker	1.09	0.75 to 1.58	0.655
Ex-smoker	1.16	0.76 to 1.79	0.490
Physical activity <30 min, 5 days/week	0.72	0.56 to 0.92	0.009
Physical activity ≥30 min, 5 days/week	0.56	0.40 to 0.78	<0.001
Age (years)	0.99	0.97 to 1.00	0.080
Eyeglasses	1.25	0.97 to 1.60	0.083
Soft contact lenses	1.31	0.90 to 1.90	0.154
Rigid contact lenses	1.34	0.56 to 3.18	0.514
Scleral contact lenses	1.45	0.63 to 3.31	0.381
Cosmetic use around the eyes	1.75	1.20 to 2.53	0.003
Refractive surgery	0.91	0.60 to 1.39	0.661
Cataract surgery	0.21	0.03 to 1.64	0.137
Glaucoma surgery	1.82	0.30 to 11.09	0.514
Strabismus surgery	1.94	0.60 to 6.30	0.272
Glaucoma	1.03	0.27 to 3.90	0.967
Facial nerve palsy	1.97	0.55 to 7.08	0.299
Hyperthyroidism	1.66	0.72 to 3.83	0.231
Hypothyroidism	0.88	0.47 to 1.64	0.677
Rheumatoid arthritis	1.01	0.51 to 1.97	0.984
Allergic rhinitis or sinusitis	1.89	1.40 to 2.56	<0.001
Autoimmune diseases	0.54	0.19 to 1.52	0.242
Asthma	1.27	0.85 to 1.90	0.249
Hypercholesterolemia	0.92	0.59 to 1.44	0.729
Diabetes mellitus	1.23	0.72 to 2.08	0.451
Hypertension	1.84	1.10 to 3.07	0.020
Depression	0.80	0.50 to 1.28	0.352
Chronic anxiety disorder	1.71	1.12 to 2.61	0.013

Table S7. Calibration of the primary model: observed against predicted risk by decile of predicted probability (n = 1,627).

Decile	Mean predicted risk	Observed risk	n
1	9.9%	8.6%	163
2	14.8%	14.7%	163
3	18.2%	17.3%	162
4	21.6%	17.8%	163
5	24.8%	28.2%	163
6	28.7%	28.4%	162
7	32.4%	32.5%	163
8	37.0%	37.0%	162
9	43.6%	54.0%	163
10	60.5%	52.8%	163
